# Trans-Kingdom Horizontal DNA Transfer from Bacteria to Yeast Is Highly Plastic Due to Natural Polymorphisms in Auxiliary Nonessential Recipient Genes

**DOI:** 10.1371/journal.pone.0074590

**Published:** 2013-09-13

**Authors:** Kazuki Moriguchi, Shinji Yamamoto, Katsuyuki Tanaka, Nori Kurata, Katsunori Suzuki

**Affiliations:** 1 Department of Biological Science, Hiroshima University, Higashi-Hiroshima, Hiroshima, Japan; 2 Plant Genetics, National Institute of Genetics, Mishima, Shizuoka, Japan; University Of Montana - Missoula, United States of America

## Abstract

With the rapid accumulation of genomic information from various eukaryotes in the last decade, genes proposed to have been derived from recent horizontal gene transfer (HGT) events have been reported even in non-phagotrophic unicellular and multicellular organisms, but the molecular pathways underlying HGT remain to be explained. The development of *in vitro* HGT detection systems, which permit the molecular and genetic analyses of donor and recipient organisms and quantify HGT, are helpful in order to gain insight into mechanisms that may contribute to contemporary HGT events or may have contributed to past HGT events. We applied a horizontal DNA transfer system model based on conjugal gene transfer called trans-kingdom conjugation (TKC) from the prokaryote *Escherichia coli* to the eukaryote *Saccharomyces cerevisiae*, and assessed whether and to what extent genetic variations in the eukaryotic recipient affect its receptivity to TKC. Strains from a collection of 4,823 knock-out mutants of *S. cerevisiae MAT-α* haploids were tested for their individual TKC receptivity. Two types of mutants, an *ssd1* mutant and respiratory mutants, which are also found in experimental strains and in nature widely, were identified as highly receptive mutants. The TKC efficiency for spontaneously accrued *petite* (*rho* ^−/0^) mutants of the functional allele (*SSD1-V*) strain showed increased receptivity. The TKC efficiency of the *ssd1Δ* mutant was 36% for bacterial conjugation, while that of the *petite*/*ssd1Δ* double mutants was even higher (220% in average) compared to bacterial conjugation. This increased TKC receptivity was also observed when other conjugal transfer systems were applied and the donor bacterium was changed to *Agrobacterium tumefaciens*. These results support the idea that the genomes of certain eukaryotes have been exposed to exogenous DNA more frequently and continuously than previously thought.

## Introduction

The transfer of genes beyond mating barriers, termed Horizontal Gene Transfer (HGT) or Lateral Gene Transfer (LGT), is now widely recognized as an important factor in bacterial evolution [1], [2]. In contrast, in eukaryotes, HGT is considered a rather limited event that mainly occurred in ancestral phagotrophic unicellular eukaryotes [3]. Previously, a cursory examination of some of the first fully sequenced eukaryotic genomes, such as the human genome, indicated presence of few, if any, genes of bacterial origin that could have been acquired by HGT [4]. However, with the rapid accumulation of genomic information from various organisms in the last decade, genes proposed to have been acquired from HGT events occurring after evolutional loss of phagotrophy have been reported even in non-phagotrophic unicellular and multicellular organisms, such as yeasts, diatoms, higher plants, and bdelloids [5]–[8]. Many of the genes predicted to have arisen by HGT in eukaryotes are considered to have bacterial origins [9].

While evidence of HGT events throughout various stages of eukaryotic evolution has accumulated, the mechanisms underlying HGT remains to be explained [5]–[9]. *In vitro* HGT detection systems have been developed for molecular and genetic analyses of donor and recipient organisms and quantification of HGT. These systems are helpful in gaining insight on mechanisms that may contribute to HGT events, both contemporary and ancient.

The type IV secretion system (T4SS) is a bacterial secretion system that transfers large DNA molecules and/or proteins. It is widely found among gram-positive and gram-negative bacteria, and its transfer capabilities extend from genetic transfer between bacterial phylums to transfer from bacteria to eukaryotes. Examples of trans-kingdom transfer by T4SS include the CagA protein-based transfer system observed in *Helicobacter pylori*, and the T-DNA transfer system of *Agrobacterium tumefaciens* that is used for gene introduction into plants [10], [11]. A bacterial conjugal transfer system, which is a type of T4SS, is encoded in the IncP-type plasmids. It has been demonstrated to be capable of transferring bacterial DNA to yeasts and mammalian cells in culture by a process referred to as trans-kingdom conjugation (TKC) [12]–[14]. In addition, the bacterial host range of this type of plasmid is promiscuous [15], which indicates that it endows donor competence on various bacteria. Based on the observed ability to facilitate DNA transfer across kingdoms and the promiscuous host range, it is conceivable that T4SS-based TKC might represent a potential driving force behind HGT from bacteria to eukaryotes.

In this study, we attempted to identify the genetic features of a recipient that enable high receptivity, especially those that are spontaneously distributed in various strains. We examined efficiency of DNA transfer from *E. coli* to various genetically distinct strains of *S. cerevisiae* by TKC carried on a common IncP1α type plasmid, RK2 (RP4). *S. cerevisiae* was chosen as the eukaryotic model for testing our hypothesis for the following reasons: (a) yeast genes predicted to have arisen from bacteria via HGT have been previously reported [7], [16], and (b) a complete collection of yeast knock-out mutants is available, which allowed systematic and comprehensive analysis of the impact of the genetic variations in the eukaryotic recipient on its receptivity in TKC.

## Materials and Methods

### Yeast and Bacterial Strains and Media

The complete set of Yeast Deletion Clones (*MAT-α* haploids complete set) was purchased from Invitrogen (Carlsbad, CA). All other yeast and bacterial strains used in this study are listed in Table 1, including those provided by the National Bio-Resource Project (NBRP) of MEXT, Japan. The media used included YPD (1% yeast extract, 2% polypeptone, 2% glucose), YPG (1% yeast extract, 2% polypeptone, 2% glycerol), and synthetic defined medium (SD, containing 0.67% yeast nitrogen base w/o amino acids, 2% glucose, and addition of appropriate individual amino acids and/or uracil) for *S. cerevisiae*, and Luria Bertani broth (LB: 1% tryptone, 0.5% yeast extract, 1% NaCl) for *E. coli* and *A. tumefaciens*. TNB (40 mM Tris-HCl (pH 7.5) and 0.5% NaCl) was used for the standard TKC reaction. Alternatively, TNB at pH 6.5, MNB (40 mM MES (pH 5.5) and 0.5% NaCl), and PBS (purchased from Becton, Dickinson and Company; adjusted to pH 7.5) were used for analyzing the adaptability of TKC under various buffers. The freshwater was collected from Yamanaka-Ike pond in Hiroshima University, and sterilized by microfiltration using DISMIC-25AS (0.2-µm pore; ADVANTEC, Tokyo, Japan). All media ingredients or media except polypeptone were purchased from Becton, Dickinson and Company (Franklin Lakes, NJ). Polypeptone and all chemicals were purchased from Wako Pure Chemical Ind. Ltd. (Osaka, Japan).

**Table 1:** Strains and plasmids used in this study.

Strains and plasmids	Relevant characteristics	Source or reference
**Strains**		
***S. cerevisiae***		
BY4742	*MAT*α *SSD1-V his3Δ1 leu2Δ0 lys2Δ0 ura3Δ0*	Invitrogen
Complete set of Yeast deletion clones	*MAT*α haploids complete set of BY4742 background. Each gene is disrupted by *kanMX4.*	Invitrogen
TKCU2000R0	BY4742 *rho* ^0^	This study
TKCU2000P4	BY4742 spontaneous petite mutant clone 4	This study
TKCU2000P7	BY4742 spontaneous petite mutant clone 7	This study
TKCU2000P8	BY4742 spontaneous petite mutant clone 8	This study
TKCU2000P9	BY4742 spontaneous petite mutant clone 9	This study
TKCU2005P1	BY4742 *ssd1Δ::kanMX4* spontaneous petite mutant clone 1	This study
TKCU2005P4	BY4742 *ssd1Δ::kanMX4* spontaneous petite mutant clone 4	This study
TKCU2005P5	BY4742 *ssd1Δ::kanMX4* spontaneous petite mutant clone 5	This study
TKCU2005P6	BY4742 *ssd1Δ::kanMX4* spontaneous petite mutant clone 6	This study
BY4741	*MAT*a *SSD1-V his3Δ1 leu2Δ0 met15Δ0 ura3Δ0*	Invitrogen
TKCU1000R0	BY4741 *rho* ^0^, used for generating diploid strains of BY4742 deletion mutants	This study
TKCU1001	BY4741 *ylr374cΔ::kanMX4*	This study
TKCU1002	BY4741 *pho85Δ::kanMX4*	This study
TKCU1003	BY4741 *far1Δ::kanMX4*	This study
TKCU1004	BY4741 *vid28Δ::kanMX4*	This study
TKCU1005	BY4741 *ssd1Δ::kanMX4*	This study
EGY48	*MAT*α *SSD1-V ura3-52 his3 trp1 GAL1p-lexA(OP)6-LEU2*	Clontech
TKCU4005	EGY48 *ssd1Δ::kanMX4*	This study
W303-1B	*MAT*α *ssd1-d ura3-1 leu2-3,112 trp1-1 his3-11,15 ade2-1 can1-100 rad5-535*	NBRP Japan
***E. coli***		
HB101	*F^−^ hsdS20(r^−^_B_ m^−^_B_) recA13 ara-14 proA2 lacY1 galK2 rpsL20 xyl-5 mtl-1 supE44 l^−^ leu thi*	NBRP Japan
Sy327 (λ*pir*)	Rif^R^ Nal^R^ λ*pir*	NBRP Japan
***A. tumefaciens***		
C58C1	pTiC58-cured and Rif^R^ derivative of C58	Yamamoto et al. [38]
**Plasmids**		
pRS313	*Saccharomyces/E. coli* shuttle vector; *HIS3 CEN6/ARSH4 ori*-pMB1 Amp^R^	NBRP Japan
pRS313::*SSD1-V*	*SSD1-V* integrated pRS313	T. Kokubo, [17]
pRS313::*ssd1-d*	*ssd1-d* integrated pRS313	T. Kokubo, [17]
pAY205	Mobilizable plasmid; *oriV* ^Q^ *oriT* ^Q^ *mob* ^Q^ *URA3 TRP1 ARS1* Kan^R^ Tet^R^	*AB526841, [39]
pYN402	Mobilizable plasmid; *oriV* ^Q^ *oriT* ^Q^ *mob* ^Q^ *URA3* 2µ*-ori* Gm^R^	*AB531984
**pRS313::*oriT* ^P^	Mobilizable plasmid; *HIS3 CEN6/ARSH4 ori*-pMB1 Amp^R^ *oriT* ^P1α^	This study
**pRS315::*oriT* ^P^	Mobilizable plasmid; *LEU2 CEN6/ARSH4 ori*-pMB1 Amp^R^ *oriT* ^P1α^	This study
**pRS316::*oriT* ^P^	Mobilizable plasmid; *URA3 CEN6/ARSH4 ori*-pMB1 Amp^R^ *oriT* ^P1α^	This study
RP4	IncP1α-type conjugative broad host range plasmid	Pansegrau et al. [40]
pRH210	Helper plasmid; *tra* ^P1α^ *trb* ^P1α^ *or*i-pMB1 Amp^R^	*AB526839, [41]
pRH220	Helper plasmid; *tra* ^P1α^ *trb* ^P1α^ *ori*-pSC101 Cm^R^	*AB526840, [42]
pDPT51	Helper plasmid; *tra* ^P1β^ *trb* ^P1β^ *ori-*ColE1 Tp^R^ Amp^R^	Y. Fujita

* DDBJ/EMBL/GenBank accession number.

** These TKC vectors were deposited to NBRP-Yeast, Japan (http://yeast.lab.nig.ac.jp/nig/index_en.html) for universal use.

### Plasmids

The plasmids used in this study are shown in Table 1. The pRS313 vector, which was used for complementation analysis, was provided by the National Bio-Resource Project (NBRP) of MEXT, Japan. The *SSD1-V* and *ssd1-d* constructs were provided by Dr. T. Kokubo [17]. The helper plasmid pDPT51 was provided by Dr. Y. Fujita (Graduate School of Bioagricultural Science, Nagoya University, Nagoya, Japan).

### Screening for High-receptivity Mutants

Cultures of the donor bacterium, *E. coli* HB101 or *A. tumefaciens* C58C1, carrying appropriate plasmids, grown in liquid medium containing appropriate antibiotics were collected, and resuspended in TNB at a concentration of 1.5×10^8^ cfu/ml. *E. coli* suspension (25 µl) containing 3.8×10^6^ cfu was used for each TKC reaction. Cultures of recipient yeast strains (parental or mutant strains) from a 96-well frozen stock plate were replica plated on YPD plates, and incubated for 48 h at 28°C. The yeast cells were picked up with toothpicks from the YPD plate, each approximately 1×10^6^ cfu, and directly suspended in the *E. coli* suspension, to obtain a donor:recipient ratio of approximately 3.8∶1. The mixed suspension was then incubated for 1 hour at 28°C (TKC reaction). This incubation was critical for achieving stable and efficient TKC because the synthetic defined medium blocked TKC. Aliquots of the suspension were plated on a yeast growth medium to select for transconjugants, and incubated for 72 h at 28°C. The selection medium used lacked uracil, but contained 30 µg/ml chloramphenicol. In the first and second rounds of screening, a volume equivalent to 20% of the TKC reaction suspension was plated on the selection plate, while in the third round of screening, 50% was plated.

TKC efficiency was measured by counting the colonies on the selection plates, and compared between the parental and mutant strains. In the first round of screening mutants showing a ≥4 fold for the ratio of TKC efficiency between the mutant and its parental strain were selected as enhanced receptivity mutants. These were subjected to repeat TKC reaction and a second round of screening, where mutants showing ≥8 fold were selected. At the third screening, the TKC reaction suspension was plated on both, the selection medium and the complete medium (YPD containing 30 µg/mL chloramphenicol), and the colonies were counted. TKC efficiency was expressed as the ratio of no. of colonies on selection medium to that on complete medium, and compared between various mutants and the parental strain. Mutants showing ≥16 fold either at the second or the third screening were defined as the high-receptivity mutants.

### Standard TKC Reaction

The standard TKC reaction on this study was performed following the method used for the third screening except that each 12.5-µl volume of *E. coli* HB101 or *A. tumefaciens* C58C1, carrying appropriate plasmids, and yeast suspension in TNB, containing 3.8×10^6^ and 1.0×10^6^ cfu respectively, which were measured and adjusted by using a spectrometer, were mixed. The scale of the reaction was increased up to 3-fold to detect transconjugants when a reaction condition was stringent for TKC. TKC efficiency determined based on recovery of uracil prototrophic transconjugants and was expressed as number of colonies on selection plate divided by the number of colonies on complete plate (YPD with 30 µg/ml chloramphenicol), adjusted by dilution ratios.

### Yeast Genetic Methods

Elimination of cytoplasmic *petite* mutants was accomplished by crossing the 22 candidate high-TKC-receptivity mutants of a *MAT*α strain were crossed with a *MAT*a strain derived from BY4741 strain that had a homologous nuclear genotype, but a *rho* ^0^ mitochondrial genotype. The resultant diploids were examined for their ability to respire (Figure S3).

In other experiments, a chemical transformation method using Lithium acetate [18] was applied for checking transformation efficiency in high TKC receptivity mutants, or *S. cerevisiae.* Direct Transformation Kit from Wako Pure Chemical Ind. Ltd. (Osaka, Japan) was used for complementation with plasmid encoded genes and for generating knock-out strains. Knock-out mutants were generated by integrating deletion cassettes containing *kanMX4*. The deletion cassette targeting each gene was prepared by PCR using a specific primer set and appropriate genomic DNA derived from the respective yeast deletion clones as the template. The primer sets used for the generation of knock-out mutants are listed in Table S3.

### Bacterial Conjugation

Standard protocols for conjugation between two bacterial strains followed the standard TKC reaction, and the transconjugants were selected on LB solid medium containing the appropriate antibiotics rifampicin (30 µg/ml) and kanamycin (50 µg/ml).

### Mitochondrial Inhibitor Treatment

**Figure 1:**
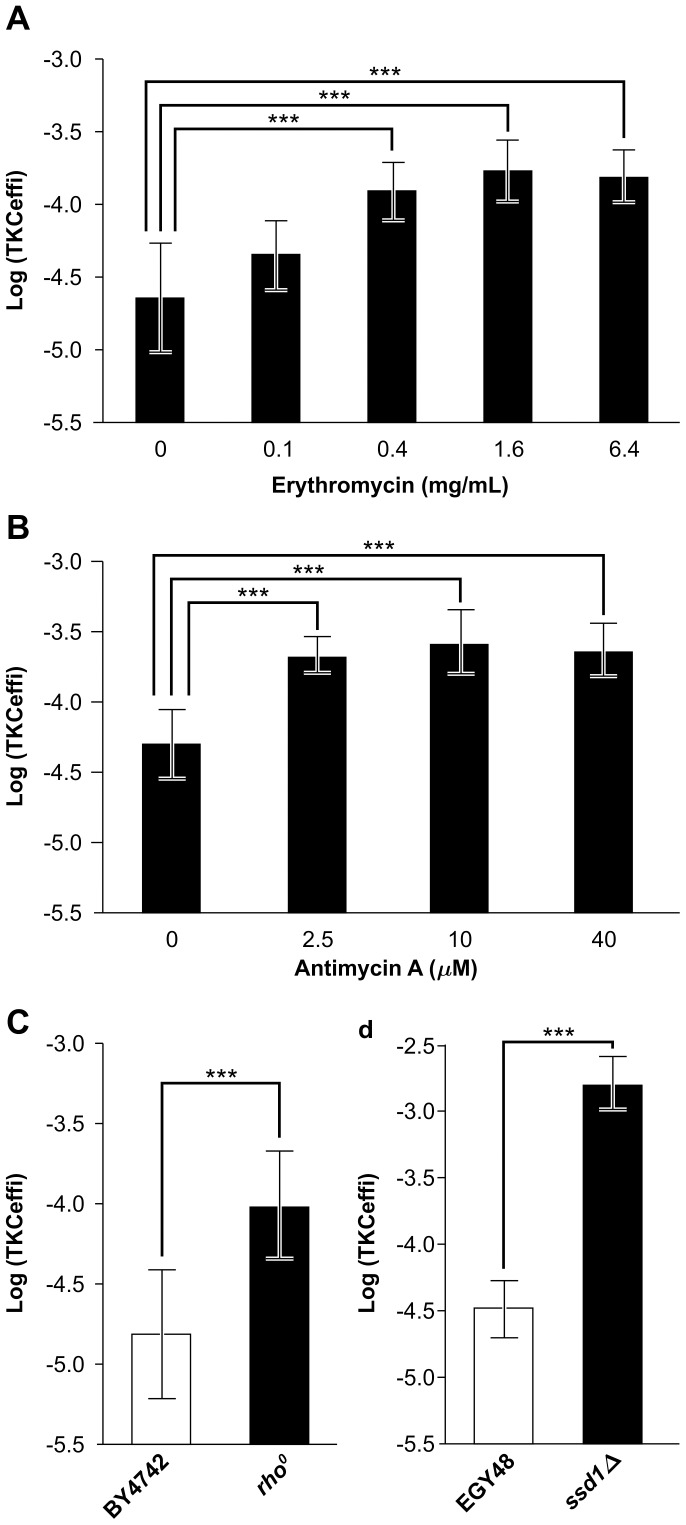
Effect of mitochondrial functional integrity and *SSD1* mutation on efficiency of DNA transfer from *Escherichia coli* HB101 to *Saccharomyces cerevisiae* strains (BY4742 in (A), (B) and (C); EGY48 in (D)) by trans-kingdom conjugation (TKC). Enhanced TKC efficiency was observed in (A) the pretreatment of the recipient yeast with a mitochondrial translational inhibitor, erythromycin; (B) the pretreatment of the recipient yeast with a mitochondrial respiration inhibitor, antimycin; (C) a *rho^0^* strain, lacking mitochondrial genome; and (D) an *SSD1-*knock-out mutant. The vertical axis “Log (TKCeffi)” represents the value of TKC efficiency (no. of transconjugant colonies/no. of recipient cell) converted to Log_10_. Data are represented as mean ± SD (n = 9 in A, B and D, 17 in C). Asterisks indicate a statistically significant difference: ***p<0.001 (two-tailed *t*-test). HB101 (pRH210, pAY205) was used as the donor.

Yeast strains were incubated overnight at 28°C on YPD plates containing either antimycin or erythromycin at various concentrations according to previous literature [19], [20] and shown in Figure 1.

### Statistical Analysis

All statistical analyses were performed using either Microsoft Excel or the public domain R program (http://www.R-project.org/).

## Results

### Identification of High-TKC-receptivity Mutants by Genome Wide Screening

TKC was measured on the basis of transfer *URA3* gene from an *E. coli* donor strain into various *ura3* ^−^ recipient yeast strains, mediated by a mobilizable plasmid pAY205, capable of replication in bacteria and yeast. The TKC was facilitated by a helper plasmid, pRH210, carrying *tra* genes for T4SS, which were derived from RP4, and was scored by the recovery of uracil autotrophic transconjugants (Figure S1). In order to create experimental conditions that more closely mimicked those of the natural environment, cells of the donor and recipient strains were resuspended in a buffer (TNB) that did not contain any chemical reagents that are known to increase cell permeability, such as polyethylene glycol and dithiothreitol (Materials and Methods, 21). TKC efficiency was determined for the complete collection of 4,823 knock-out mutants derived from parental strain, *S. cerevisiae* BY4742. We identified 22 high-receptivity mutants (Table S1), defined as having 16-fold or greater TKC efficiency as compared with parental strain. These mutants differed from yeast mutants that have been previously identified as having a greater efficiency in chemical DNA transformation than the wild-type strains [21]. These mutants did not show obvious increase in transformation efficiency when a chemical yeast transformation method was applied using the same vector, pAY205 (Table S1).

### Deficiency in Mitochondrial Function Induces High TKC Receptivity

Because 8 out of the 22 high-receptivity yeast strains were mutants for known, nuclear-encoded mitochondrial genes, and numerous additional strains classified as high-receptivity mutants albeit at a lower threshold (<16-fold at 2^nd^ and 3^rd^ screenings; see Materials and Methods) were also mutants in nuclear-encoded mitochondrial genes (Figure S2), we surmised that the functional integrity of the mitochondrion might serve as a means for blocking TKC.

Indeed, treatment of the parental strain with drugs that inhibit mitochondrial translation (erythromycin) or respiration (antimycin A) caused an increase in TKC levels by approximately 7- or 5-fold, respectively (Figure 1A and 1B). In addition, increase in TKC relative to wild-type strains was also observed for *rho* ^0^ mutants lacking mitochondrial DNA (Figure 1C). To determine whether the increase in TKC in all of the remaining 14 yeast mutant strains was also due to deficiencies in mitochondrial function resulting from either primary mutations in targeted genes within the deletion mutant collection or secondary cytoplasmic *petite* mutants [22] that are frequently and spontaneously produced from yeast strains and form small (“*petite*” in French) colonies based on their defective phenotype of respiratory function, we screened these strains for mitochondrial deficiency by crossing with a *rho* ^0^ mutant. Nine mutants out of the 14 were cytoplasmic *petite* mutants (Figure S3).

### *SSD1*, a Polymorphic Locus, Strongly Influences TKC Receptivity

The remaining 5 (*far1Δ, pho85Δ, ssd1Δ, vid28Δ*, and *ylr374cΔ*) out of the 14 mutant strains proved capable of respiration, indicating that while the respective mutations increased TKC efficiency compared to wild-type yeast, they did not affect minimal mitochondrial function or cell viability. To further implicate the mutations in the identified genes with the observed increase in TKC, not caused by additional mutations in other loci in the 5 mutant strains, TKC efficiencies were also determined for knock-out mutants of the 5 respective genes generated in another yeast parental strain BY4741, and a mutant lacking the suppressor of *SIT4* deletion 1 (*SSD1*) gene showed high receptivity (Figure S4A). The highest increase in TKC (74.9-fold increase over the parental strain at the 3^rd^ screening) was observed in yeast recipient strains with a mutation in *SSD1* (Table S1), a nonessential gene encoding an RNA-binding protein [23], the deficiency of which alters cell wall composition in various fungi [24]–[28]. Our results indicated that the *SSD1* appears to play a major role in blocking TKC from *E. coli* to yeast (Figure 1D and 4, Table S1). Interestingly, the *SSD1* is a polymorphic locus [29], [30] with one allele, *SSD1-V*, producing a full-length Ssd1 protein, and another allele, *ssd1-d*, producing a truncated protein terminating at the beginning of its RNA-binding domain due to a nonsense mutation [31], [32].

**Figure 2:**
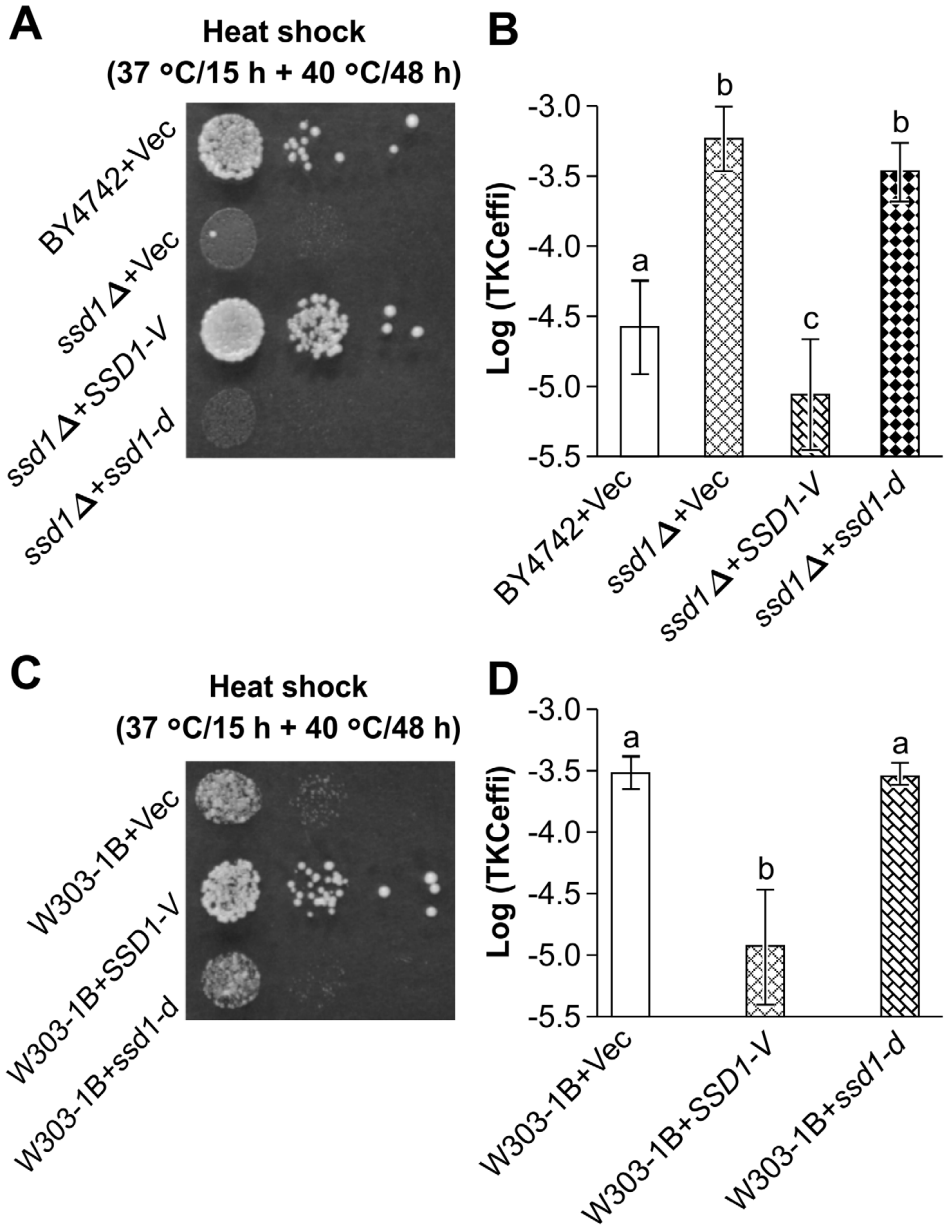
Functional complementation of *SSD1* mutation rescues its temperature sensitivity as well as high TKC receptivity phenotype. *Saccharomyces cerevisiae* strains carrying a deletion (BY4742 *ssd1Δ*) or a strain spontaneously carrying the truncated *SSD1* (W303-1B) was transformed with either the vector alone (vec), or vector carrying full length (*SSD1-V*) or vector carrying truncated (*ssd1-d*) genes. The growth phenotype on solid media at 37°C for 15 h followed by 42°C for 48 h with serial dilution (A) and (C); and the DNA transfer efficiency by the standard TKC procedure was measured (B) and (D). Complementation with the full length, functional *SSD1*, but not the truncated gene rescued the phenotypes tested. Data are represented as mean ± SD (n = 15 in B and 6 in D, respectively). Letters above the bars indicate significant differences at *p*<0.001 (Holm’s test). HB101 (pRH210, pAY205) was used as the donor.

To further clarify the blocking function of *SSD1*, the relationship between the functional integrity of the *ssd1-d* allele on TKC was investigated. A known phenotype of *SSD1* deficiency is temperature sensitivity, and this was observed in both the *ssd1Δ* and *ssd1-d* mutant strains [32] (Figure 2A and 2C). Complementation analysis with the *SSD1-V* allele was shown to rescue temperature sensitivity while simultaneously rescuing the TKC blocking-deficient phenotype of the *ssd1Δ* mutant (Figure 2A and 2B) and an *ssd1-d* strain W303-1B (Figure 2C and 2D); however, complementation of the *ssd1-d* allele did not rescue these phenotypes in both the *ssd1Δ* (Figure 2A and 2B) and *ssd1-d* (Figure 2C and 2D) strains. Thus, polymorphism in the *SSD1* gene in the recipient yeast strain influenced TKC-based DNA receptivity.

### Characterization of High Receptivity Mutants

**Figure 3:**
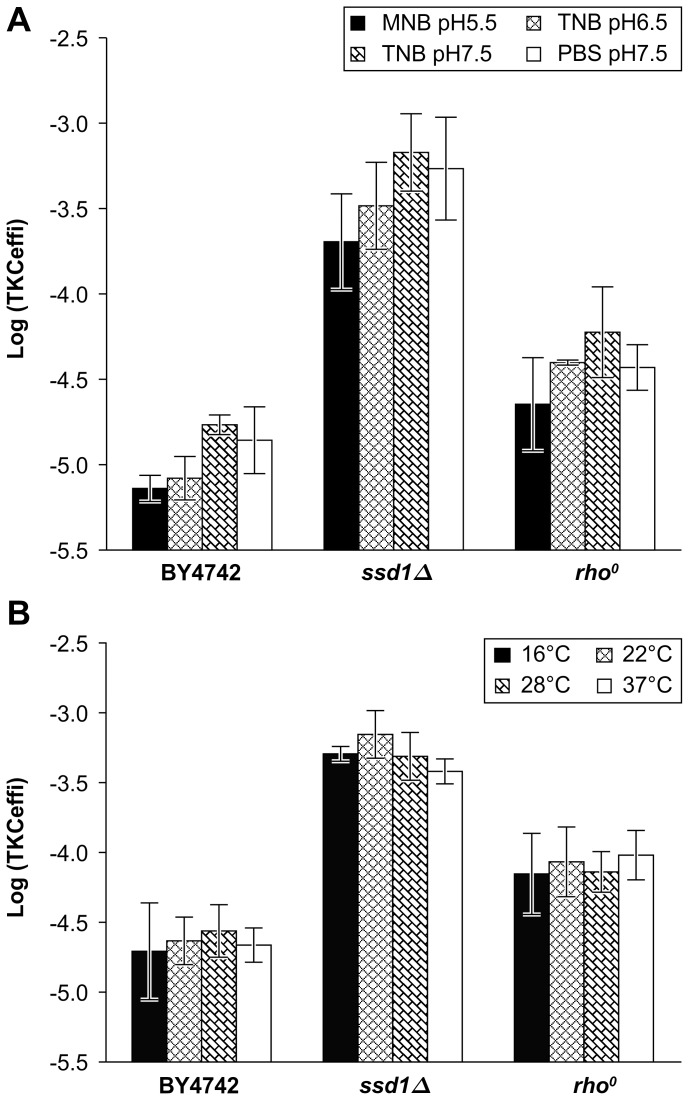
Universality of high receptivity under various reaction conditions in high receptivity mutants. TKC efficiency was examined using *SSD1* deletion mutant (*ssd1Δ*), and *rho^0^* mutant in various reaction buffers (A) or under various temparature (B). Data are represented as mean ± SD (n = 3). In all reaction conditions examined, the high recepitivity mutants showed higher recepitivity comparing with parental strain, and they were statistically significant at *p*<0.05 except *rho^0^* mutant at 16°C (two-tailed *t*-test). HB101 (pRH210, pAY205) was used as the donor.

As it is expected that *SSD1* and petite mutants can be normally distributed in natural, industrial, and laboratory environments, we focused on their high receptivity. No uracil autotrophic yeast was observed when the mutant recipients were exposed to the donor *E. coli* without helper plasmid (Table S2). Since the helper plasmid encodes *tra* genes, which are essential for TKC (Figure S1), this result confirmed that the recovered yeasts found in increased amounts in highly receptive mutants were indeed transconjugants resulting from TKC, not transformants resulting from the increased direct incorporation of the reporter plasmid DNA from lysed donors. TKC reactions were also performed under various physiological conditions by changing the reaction buffer and temperature. The high receptivity in mutants was observed at the same order of efficiency compared with the standard condition (TNB: pH7.5, 28°C), although they showed decreased receptivity along with the parental strain at lower pH (pH5.5; Figure 3A) and lower temperature (16°C; Figure 3B). The temperature sensitive mutant, *ssd1Δ* strain, showed highest TKC efficiency at 22°C and lowest at 37°C (Figure 3B). We further examined TKC using freshwater and compared its efficiency with bacterial conjugation using the identical DNA transfer system mediated by the plasmids pAY205 and pRH210 (Table 1). Decreased transfer efficiency was observed in the transfer from *E. coli* (HB101) to *S. cerevisiae* (parental strain and the mutants) and to *A. tumefaciens* (C58C1), but not to *E. coli* (Sy327), in comparison with the transfer efficiency using TNB (Figure S4B). Interestingly, the receptivity of *ssd1Δ* mutants was higher that of *E. coli*, and comparable with that of *A. tumefaciens* in freshwater (Figure S4B). The receptivity of *rho* ^0^ mutants was also comparable with that of *E. coli*. These data suggested that there might be naturally existing yeast strains that are as receptive to DNA transfer from bacteria as are other bacteria.

**Figure 4:**
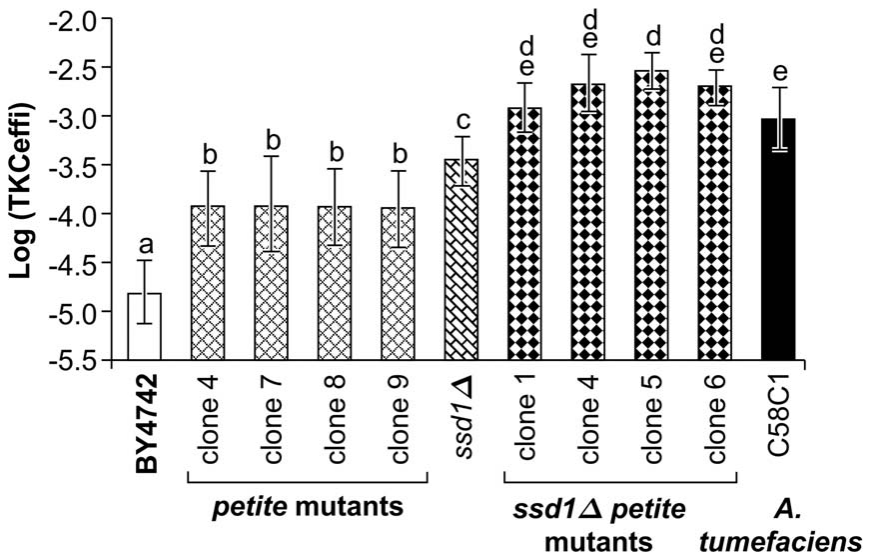
Efficiency of DNA transfer by TKC between a bacterial strain and yeast can be comparable to that for conjugation between two bacterial strains. TKC reactions were set up between *Escherichia coli* HB101 and *Saccharomyces cerevisiae* parental strain BY4742, or its petite mutants (clones 4–9), or a *SSD1* deletion mutant (*ssd1Δ*), or petite mutants of the *SSD1* deletion mutant (clones 1–6). The DNA transfer efficiencies of the above TKC were compared with conjugation between the same strain of *Escherichia coli* HB101 and *Agrobacterium tumefaciens* C58C1. Data are represented as mean ± SD (n = 15 in BY4742, *ssd1Δ* and C58C1, n = 9 in others). Letters above the bars indicate significant differences at *p*<0.05 (Holm’s test). HB101 (pRH210, pAY205) was used as the donor.

In order to investigate the relationship between *SSD1* and mitochondrial mutations, the TKC efficiencies of *petite/ssd1Δ* double mutants were compared to those of petite or *ssd1Δ* single mutant strains (Figure 4). Secondly, the various TKC efficiencies for DNA transfer between *E. coli* and the yeast strains were also compared to that for the interspecies conjugation between *E. coli* and another bacterium, *A. tumefaciens*, using an identical DNA transfer system mediated by plasmids pAY205 and pRH210. The average TKC efficiency for *petite* mutants or the *SSD1-V* strain was 6-fold lower than the conjugation efficiency between the two bacterial species, whereas the TKC efficiency for the *ssd1Δ* mutant was 36% of that for bacterial conjugation (Figure 4). Interestingly, the average transfer efficiency in the *petite*/*ssd1Δ* double mutants was even higher than that between the 2 bacterial species (Figure 4). The synergistic increase in TKC efficiency upon simultaneous loss of mitochondrial function and *ssd1Δ* as compared to that in the parental strain or the single mutant strains indicates that the different mutations affected pathways that are independent or at least partly distinct. These data also support the notion that there may be naturally existing yeast strains that are as receptive to DNA transfer from bacteria as are other bacteria.

Because deficiency of *SSD1* alters cell wall composition in various fungi [24]–[28], we supposed that it led to an increased attachment between the donor and host cells and/or an increased permeability via T4SS. Thus, the parental, and *ssd1Δ* and *rho* ^0^ mutant recipient yeast strains were treated with cell wall-degrading enzymes prior to the DNA transfer process. The cell wall digestion treatment had a negative effect on the overall level of TKC as compared to the untreated or mock-treated yeast cells of the same strain (Figure S4C); however, it did not affect the relative differences in TKC efficiency between the *ssd1Δ* mutant and the parental strain (Figure S4C). This result suggests that increased DNA receptivity in *ssd1Δ* might be mediated by factors other than the cell wall attachment and/or permeability.

### Receptivity in Various TKC Transfer Processes

**Figure 5:**
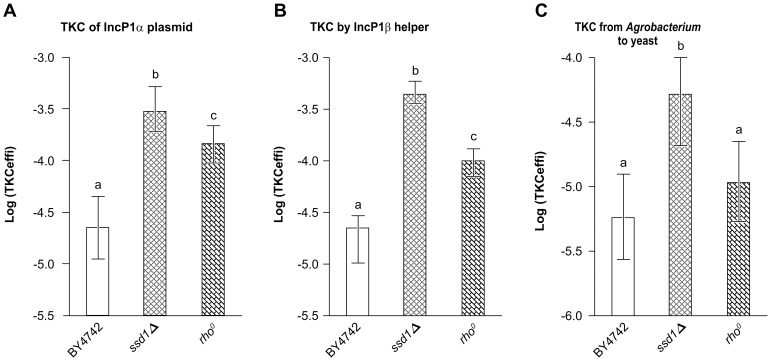
Universality of components comprising TKC between a bacterial strain and a high-DNA-receptivity yeast mutant strain. Efficiency if DNA transfer was measured in the parental yeast strain or its high-receptivity mutants, *SSD1* deletion mutant (*ssd1Δ*), and *rho^0^* mutant, after TKC with *Escherichia coli* HB101 (pRH220, pRS316::*oriT* ^P^) carrying T4SS functions on IncP1α (A), or with *Escherichia coli* HB101 (pDPT51, pAY205) carrying T4SS functions on IncP1β plasmid (B), or with *A. tumefaciens* C58C1 (RP4, pYN402) (C). Data are represented as mean ± SD (n = 9 in A and C, n = 15 in B). Letters above the bars indicate significant differences at *p*<0.01 in A, 0.001 in B, and 0.001 in C (Holm’s test).

In the experiments described so far, the TKC process was mediated by an IncP1α plasmid encoding T4SS, and the mobilizable plasmid pAY205 derived from a broad host-range IncQ plasmid, RSF1010. We investigated whether the observed high receptivity of *ssd1Δ* and *petite* mutants was specifically linked to the IncQ plasmid-based transfer or would be observed with other mobilizable plasmids. Hence, TKC efficiency using an IncP1α mobilizable plasmid, pRS316::*oriT* ^P^ was determined. The results demonstrate that the TKC efficiency of the *ssd1Δ* mutant was >12-fold higher and that of the *rho* ^0^ *petite* mutant was 5-fold higher than that of the corresponding parental strain (Figure 5A). The increased TKC efficiency was also observed when other vectors, having *HIS3* or *LEU2* instead of *URA3*, were used (Figure S5). Next, the TKC efficiency was determined using T4SS encoded on a helper plasmid, pDPT51, derived from the IncP1β plasmid, R751, which has been shown to induce TKC efficiently [12]. Remarkably, the TKC efficiency of both the *ssd1Δ* and *rho* ^0^ mutants increased to the same level as that observed when using the IncP1α helper plasmid. The receptivity of the *ssd1Δ* mutant was >30-fold higher, while that of the *rho* ^0^ mutant was >6-fold higher than that of the corresponding parental strain (Figure 5B). These data indicate that the high TKC DNA receptivity caused by the defects in the polymorphic *SSD1* gene and mitochondrial genes can be widely applied, and is not limited to the IncQ plasmid transfer mediated by T4SS encoded by the IncP1α plasmid.

Lastly, the DNA transfer efficiency from another bacterium, *A. tumefaciens*, to yeast was determined. An *A. tumefaciens* strain carrying a wild IncP1α plasmid, RP4, which is the parental plasmid of pRH210, and an IncQ-type mobilizable plasmid, pYN402, derived from RSF1010 was used as the donor strain, and the various yeast strains were used as recipients. IncP plasmids have a broad host range among proteobacteria [15], as well as a broad transfer range. The receptivity of the *ssd1Δ* mutant was over 8-fold higher than that of the parental strain; however, the basal efficiency of DNA transfer from *A. tumefaciens* to yeast strains was lower than that from *E. coli* by one order of magnitude (Figure 5C). In the *rho* ^0^ mutant, receptivity was slightly higher than in the parental strain, although the difference was not statistically significant (Figure 5C). Thus, the observed increase in receptivity of yeast mutants was not limited to DNA transfer from *E. coli*, suggesting that TKC may be more universal and widespread in nature than previously indicated and that T4SS-based TKC is a possible driving force underlying the transfer of genes from various bacteria to ascomycete yeast and potentially other eukaryotes.

## Discussion

**Figure 6:**
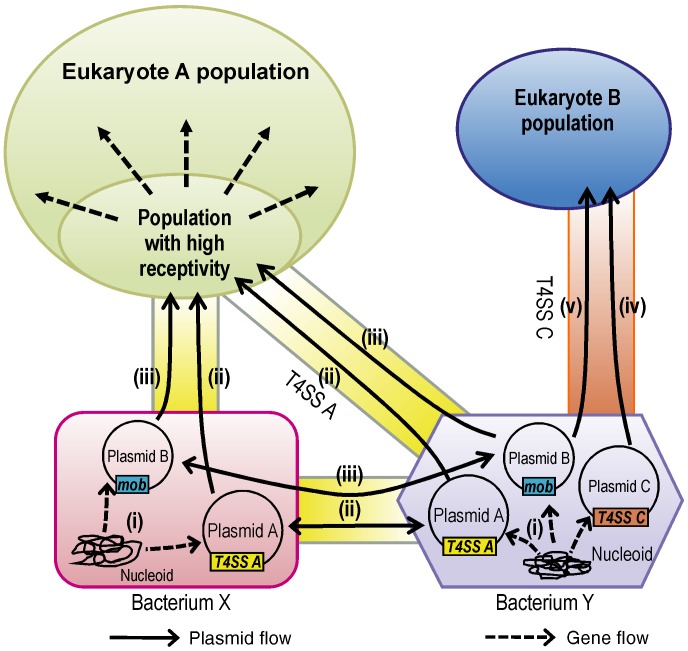
A schematic diagram of the gene and plasmid flow from bacteria to eukaryotes by T4SS. In bacteria X and Y, genes are transferred from genomic DNA to various plasmids depicted as A, B, and C by using transposons or another method (i). A conjugative plasmid A (such as IncP plasmids) can move from bacterium X to another bacterium Y or to a eukaryote A by its own T4SS system, i.e., T4SS A (ii). A mobilizable plasmid B (such as IncQ plasmids) transfers from bacterium X to Y and eukaryote A with the help of its own *mob* genes and T4SS A (iii). The plasmids A and B can also transfer from bacterium Y to eukaryote A by T4SS A (ii and iii). The eukaryote A has a subpopulation that accepts exogenous DNA efficiently transferred by T4SS A. The transferred bacterial genes spread and are maintained in the entire population, if they take on available roles, for its survival. In bacterium Y, a plasmid C transfers to eukaryote B by T4SS C (such as *vir* genes in Ti and Ri plasmids; iv). The plasmid B is also able to transfer to eukaryote B by using *mob* genes and T4SS C (v).

Alterations in the genetic makeup are integral to evolution of species. Our results reveal two important features regarding the contribution of HGT to eukaryotic evolution: (1) the receptivity of a recipient eukaryotic organism to DNA transfer from a bacterium may be altered drastically by the presence of different alleles of certain loci and by the polymorphisms in non-essential genes, which are not primarily coded for blocking HGT in the recipient eukaryote, and (2) T4SS-mediated TKC may represent the mechanistic means behind HGT from bacteria to eukaryotes (Figure 6).

Yeast mutants such as the *petite* (*rho* ^−/0^) mutants studied here that exhibit defects in mitochondrial function through a partial or complete lack of mitochondrial DNA comprise approximately 1–2% of natural yeast populations [22]. Petite mutant accumulation can be increased even further, e.g., by other mutations such as a single nucleotide polymorphism (SNP) in the so-called *MIP1* gene that can increase petite mutant accumulation by 4-fold [33]. SNPs in *SSD1* have also been found in various wild and clinically isolated yeast strains used in the Saccharomyces Genome Resequencing Project (SGRP; 34). Eighteen non-synonymous SNPs in *SSD1* have been found, with two of them being nonsense SNPs [34]. Although the growth or survival advantage of the existence of various SNPs, including defective mutations, in these genes is unclear, it is clear that mutants receptive to DNA via TKC emerge frequently and are widely distributed in nature. In addition, the TKC efficiency of high-receptivity strains was comparable with the conjugation efficiency from *E. coli* to *A. tumefaciens* or *E. coli* under certain conditions (Figures 4 and S4C). TKC may be one of the major driving forces behind gene transfer from bacteria to eukaryotes under natural conditions. The identification of mutants in nonessential genes with higher TKC efficiency indicates the possibility that various subpopulations within recipient species can accept exogenous DNA at higher rates (Figure 6). The existence of such a subpopulation may be applied not only to TKC but also to other DNA transfer or HGT mechanisms that might exist.

Due to the lack of experimental approaches, past HGT events have thus far been deduced mainly from *in silico* analyses. Even in the case of T-DNA transfer, which continuously occurs in nature and fields, the past T-DNA transfer events have only been found in genomes of one genus (*Nicotiana*) [35]. Thus, it is very difficult to identify direct evidence that can prove the pathway of ancient HGT events.

For these reasons, the establishment of *in vitro* HGT detection systems that enable molecular and genetic analyses of donor and recipient organisms as well as the quantification of HGT–such as a TKC model system–would be helpful for elucidating the mechanisms that contribute to HGT events both contemporary and past events. In the model HGT system for DNA transfer from bacteria to yeast designed and presented here, we emphasize that the TKC process was carried out in a suspension of donor and recipient cells under ambient conditions (Figures 3 and S4B), in the absence of heat shock step and/or membrane destabilizing agents, i.e. conditions that are in contrast to those applied in other methods of gene transfer in yeast [21], [36]. Such a method should be considered for its biosafety in biotechnological applications, as has been considered for bacterial conjugal transfer, which occurs in the natural environment.

Although we have shown that T4SS mediated HGT could be a convincing driving force, T4SS is not omnipotent for explaining HGT in Eukaryotes. HGT-derived genes found in bdelloid rotifers appear to have originated from various organisms such as bacteria, yeasts and plants [6], and some of the HGT-derived genes reported in higher plants are likely to have originated from fungi [8]. Again, these data strongly indicate the importance of experimental approaches that focuses on the driving force of HGT in addition to phagotrophy and TKC.

A recent study showed that TKC may also be useful and applicable as a method for introducing bacterial genes into eukaryotes [37]. Its main advantage is the simplicity of execution. The only requirement is of generating an *E. coli* strain with a helper plasmid and a plasmid encoding the gene of interest to be transferred. Such a strain would be allowed to interact with the eukaryotic recipient to achieve HGT. The method avoids DNA extraction or having to transfer it into agrobacteria. As for *S. cerevisiae*, the treatment with antibiotics, which inhibit mitochondrial functional integrity, could easily increase TKC efficiency (Figure 1A and 1B). In addition, the EGY48 strain, which is used for the yeast two-hybrid system, was successfully modified as a high receptivity strain by introducing a mutation into *SSD1* (Figure 1D). These data show that this has potential for use as a gene introduction method. Three TKC vectors, pRS313::*oriT* ^P^, pRS315::*oriT* ^P^, and pRS316::*oriT* ^P^ were designed in such a way that their respective multiple cloning sites as well as their parental yeast shuttle vectors could be used. These vectors are available at a public bioresource bank for universal use (Table 1).

In this study, we focused on determining the potential of TKC as a driving force behind HGT and attempted to identify the genetic background that enables high receptivity in the recipient organism. Our results strongly support the idea that genomes of certain eukaryotes have been exposed to exogenous DNA more frequently and continuously than previously thought, with DNA and gene transfer frequencies from bacteria similar to those measured between prokaryotes.

## Supporting Information

Figure S1 **Schematic representation of TKC detection.** The donor *E. coli* has a helper plasmid and a mobilizable plasmid. The helper plasmid contains T4SS genes derived from an IncP1α plasmid, called *tra* genes (gray box). The mobilizable plasmid contains the origin of transfer (*oriT*; black dot) of an IncQ plasmid and *URA3* gene (black box). When the mobilizable plasmid transfers into the recipient yeast cell lacking the *URA3* gene, the transconjugant survives on selection medium with no added uracil.
(TIF)

Figure S2 **An example of the TKC results at the third screening.** (A) Template showing format of plating of the various mutants. Mutants for nuclear-encoded mitochondrial genes are underlined. * KO mutant of dubious ORF unlikely to encode a protein, and its overlapping gene is shown in parentheses. ** High-receptivity mutants included in Table S1. (B) Transconjugants grown on a selection plate; volume equivalent to 50% of each TKC reaction mix was plated. (C) Recipient yeast cells grown on a YPD+chloramphenicol plate; a volume equivalent to 1/5000 of each TKC reaction mix was plated. HB101-containing plasmids pRH210 and pAY205 were used as the donors.
(TIF)

Figure S3 **Confirmation of the mitochondrial integrity among the identified high-receptivity mutants.** The high-receptivity mutants were mated with a *MAT*a strain, carrying wild-type nuclear genome but a *rho* ^0^ mitochondrial genome derived from the BY4741 strain. The resultant heterogeneous diploid strains were serially diluted and spotted on both rich glucose (YPD) and rich respiratory glycerol (YPG) media, and were incubated at 28°C for 48 h and 72 h, respectively. The 14 KO mutants for non-mitochondrial genes are underlined.
(TIF)

Figure S4 **Confirmation and characterization of the high-receptivity mutants.** (A) Effect of knock-out mutation in the 5 candidate genes in the yeast strain BY4741 on TKC. Data are represented as mean ± SD (n = 3). Asterisks indicate a statistically significant difference: ***p<0.001 (two-tailed *t*-test). (B) Comparison between TKC and bacterial conjugation efficiency under TNB and filter-sterilized freshwater environments. Data are represented as mean ± SD (n = 5). Upper- and lowercase letters above the bars indicate significant differences at *p*<0.05 (Holm’s test) among yeast and bacterial strains under each condition. (C) Effect of cell wall digestion on TKC efficiency. Normal: the recipient cells were resuspended in TNB before testing for TKC. Mock: cells pretreated with TNB +1 M sorbitol, and TKC reaction performed in the same solution. Zymolyase: pretreatment with TNB +1 M sorbitol +0.5 mg/mL Zymolyase-100 T, and TKC reaction performed in TNB +1 M sorbitol. Each pretreatment was performed for 1 h at 28°C. Data are represented as mean ± SD (n = 7 in BY4742 with sorbitol, n = 4 in others). Lowercase letters above the bars indicate significant differences at *p*<0.05 (Holm’s test). HB101 (pRH210, pAY205) was used as the donor in all experiments.
(TIF)

Figure S5 **Confirmation of high TKC receptivity in** ***ssd1Δ*** **and** ***rho*** **^0^ mutants using other selection markers.** (A) A TKC vector pRS313::*oriT* ^P^, carrying *HIS3* gene as a selection marker, was used and the transconjugants in parental and mutant strains were selected on a selection medium plate lacking leucine. (B) A TKC vector pRS315::*oriT* ^P^, carrying *HIS3* gene as a selection marker, was used.
(TIF)

Table S1 **List of high-receptivity mutants screened from the complete set of Yeast Deletion Clones (** ***MATα*** **haploids complete set).** Efficiency of transfer of the *URA3* marker gene from *Escherichia coli* to various yeast deletion strains was measured relative to transfer to the parental strain (fold increase vs. wt).
(DOC)

Table S2 **Results of the TKC experiment without the helper plasmid.**
(DOC)

Table S3 **PCR primers used in this study.**
(DOC)

## References

[1] Koonin EV, Makarova KS, Aravind L (2001) Horizontal gene transfer in prokaryotes: quantification and classification. Annu Rev Microbiol 55: 709–742. 11544372 10.1146/annurev.micro.55.1.709 PMC4781227

[2] Ochman H, Lawrence JG, Groisman EA (2000) Lateral gene transfer and the nature of bacterial innovation. Nature 405: 299–304. 10830951 10.1038/35012500

[3] Kurland CG, Collins LJ, Penny D (2006) Genomics and the irreducible nature of eukaryote cells. Science 312: 1011–1014. 16709776 10.1126/science.1121674

[4] Stanhope MJ, Lupas A, Italia MJ, Koretke KK, Volker C, et al (2001) Phylogenetic analyses do not support horizontal gene transfers from bacteria to vertebrates. Nature 411: 940–944. 11418856 10.1038/35082058

[5] Bowler C, Allen AE, Badger JH, Grimwood J, Jabbari K, et al (2008) The Phaeodactylum genome reveals the evolutionary history of diatom genomes. Nature 456: 239–244. 18923393 10.1038/nature07410

[6] Gladyshev EA, Meselson M, Arkhipova IR (2008) Massive horizontal gene transfer in bdelloid rotifers. Science 320: 1210–1213. 18511688 10.1126/science.1156407

[7] Hall C, Brachat S, Dietrich FS (2005) Contribution of horizontal gene transfer to the evolution of Saccharomyces cerevisiae. Eukaryot Cell 4: 1102–1115. 15947202 10.1128/EC.4.6.1102-1115.2005 PMC1151995

[8] Richards TA, Soanesb DM, Fosterc PG, Leonarda G, Thornton CR, et al (2009) Phylogenomic analysis demonstrates a pattern of rare and ancient horizontal gene transfer between plants and fungi. Plant Cell 21: 1897–1911. 19584142 10.1105/tpc.109.065805 PMC2729602

[9] Keeling PJ, Palmer JD (2008) Horizontal gene transfer in eukaryotic evolution. Nat Rev Genet 9: 605–618. 18591983 10.1038/nrg2386

[10] Cascales E, Christie PJ (2003) The versatile bacterial type IV secretion systems. Nat Rev Microbiol 1: 137–149. 15035043 10.1038/nrmicro753 PMC3873781

[11] Fronzes R, Christie PJ, Waksman G (2009) The structural biology of type IV secretion systems. Nat Rev Microbiol 7: 703–714. 19756009 10.1038/nrmicro2218 PMC3869563

[12] Heinemann JA, Sprague GF Jr (1989) Bacterial conjugative plasmids mobilize DNA transfer between bacteria and yeast. Nature 340: 205–209. 2666856 10.1038/340205a0

[13] Mizuta M, Satoh E, Katoh C, Tanaka K, Moriguchi K, et al (2012) Screening for yeast mutants defective in recipient ability for transkingdom conjugation with Escherichia coli revealed importance of vacuolar ATPase activity in the horizontal DNA transfer phenomenon. Microbiol Res 167: 311–316. 22169356 10.1016/j.micres.2011.10.001

[14] Waters VL (2001) Conjugation between bacterial and mammalian cells. Nat Genet 29: 375–376. 11726922 10.1038/ng779

[15] Schmidhauser TJ, Helinski DR (1985) Regions of broad-host-range plasmid RK2 involved in replication and stable maintenance in nine species of gram-negative bacteria. J Bacteriol 164: 446–455. 4044529 10.1128/jb.164.1.446-455.1985 PMC214264

[16] Gojkovic Z, Knecht W, Zameitat E, Warneboldt J, Coutelis JB, et al (2004) Horizontal gene transfer promoted evolution of the ability to propagate under anaerobic conditions in yeasts. Mol Genet Genomics 271: 387–393. 15014982 10.1007/s00438-004-0995-7

[17] Ohyama Y, Kasahara K, Kokubo T (2010) Saccharomyces cerevisiae Ssd1p promotes CLN2 expression by binding to the 5′-untranslated region of CLN2 mRNA. Genes Cells 15: 1169–1188. 20977549 10.1111/j.1365-2443.2010.01452.x

[18] Ito H, Fukuda Y, Murata K, Kimura A (1983) Transformation of intact yeast cells treated with alkali cations. J Bacteriol 153: 163–168. 6336730 10.1128/jb.153.1.163-168.1983 PMC217353

[19] Lefebvre-Legendre L, Balguerie A, Duvezin-Caubet S, Giraud MF, Slonimski PP, et al (2003) F1-catalysed ATP hydrolysis is required for mitochondrial biogenesis in Saccharomyces cerevisiae growing under conditions where it cannot respire. Mol Microbiol 47: 1329–1339. 12603738 10.1046/j.1365-2958.2003.03371.x

[20] Rodeheffer MS, Shadel GS (2003) Multiple interactions involving the amino-terminal domain of yeast mtRNA polymerase determine the efficiency of mitochondrial protein synthesis. J Biol Chem 278: 18695–18701. 12637560 10.1074/jbc.M301399200 PMC2606056

[21] Kawai S, Pham TA, Nguyen HT, Nankai H, Utsumi T, et al (2004) Molecular insights on DNA delivery into Saccharomyces cerevisiae. Biochem Biophys Res Commun 317: 100–107. 15047153 10.1016/j.bbrc.2004.03.011

[22] Bernardi G (1979) Petite Mutation in Yeast. (Translated from English). Trends Biochem Sci 4: 197–201.

[23] Uesono Y, Toh-e A, Kikuchi Y (1997) Ssd1p of Saccharomyces cerevisiae associates with RNA. J Biol Chem 272: 16103–16109. 9195905 10.1074/jbc.272.26.16103

[24] Gank KD, Yeaman MR, Kojima S, Yount NY, Park H, et al (2008) SSD1 is integral to host defense peptide resistance in Candida albicans. Eukaryot Cell 7: 1318–1327. 18515753 10.1128/EC.00402-07 PMC2519774

[25] Ibeas JI, Yun DJ, Damsz B, Narasimhan ML, Uesono Y, et al (2001) Resistance to the plant PR-5 protein osmotin in the model fungus Saccharomyces cerevisiae is mediated by the regulatory effects of SSD1 on cell wall composition. Plant J 25: 271–280. 11208019 10.1046/j.1365-313x.2001.00967.x

[26] Lee H, Damsz B, Woloshuk CP, Bressan RA, Narasimhan ML (2010) Use of the plant defense protein osmotin to identify Fusarium oxysporum genes that control cell wall properties. Eukaryot Cell 9: 558–568. 20190074 10.1128/EC.00316-09 PMC2863404

[27] Tanaka S, Ishihama N, Yoshioka H, Huser A, O’Connell R, et al (2009) The Colletotrichum orbiculare SSD1 mutant enhances Nicotiana benthamiana basal resistance by activating a mitogen-activated protein kinase pathway. Plant Cell 21: 2517–2526. 19706796 10.1105/tpc.109.068023 PMC2751964

[28] Wheeler RT, Kupiec M, Magnelli P, Abeijon C, Fink GR (2003) A Saccharomyces cerevisiae mutant with increased virulence. Proc Natl Acad Sci U S A 100: 2766–2770. 12589024 10.1073/pnas.0437995100 PMC151415

[29] Phatnani HP, Jones JC, Greenleaf AL (2004) Expanding the functional repertoire of CTD kinase I and RNA polymerase II: novel phosphoCTD-associating proteins in the yeast proteome. Biochemistry 43: 15702–15719. 15595826 10.1021/bi048364h PMC2879061

[30] Sutton A, Immanuel D, Arndt KT (1991) The SIT4 protein phosphatase functions in late G1 for progression into S phase. Mol Cell Biol 11: 2133–2148. 1848673 10.1128/mcb.11.4.2133-2148.1991 PMC359901

[31] Jorgensen P, Nelson B, Robinson MD, Chen Y, Andrews B, et al (2002) High-resolution genetic mapping with ordered arrays of Saccharomyces cerevisiae deletion mutants. Genetics 162: 1091–1099. 12454058 10.1093/genetics/162.3.1091 PMC1462329

[32] McDonald HB, Helfant AH, Mahony EM, Khosla SK, Goetsch L (2002) Mutational analysis reveals a role for the C terminus of the proteasome subunit Rpt4p in spindle pole body duplication in Saccharomyces cerevisiae. Genetics 162: 705–720. 12399382 10.1093/genetics/162.2.705 PMC1462277

[33] Baruffini E, Lodi T, Dallabona C, Foury F (2007) A single nucleotide polymorphism in the DNA polymerase gamma gene of Saccharomyces cerevisiae laboratory strains is responsible for increased mitochondrial DNA mutability. Genetics 177: 1227–1231. 17720904 10.1534/genetics.107.079293 PMC2034627

[34] Saccharomyces Genome Resequencing Project, http://www.sanger.ac.uk/research/projects/genomeinformatics/browser.html.

[35] Suzuki K, Yamashita I, Tanaka N (2002) Tobacco plants were transformed by Agrobacterium rhizogenes infection during their evolution. Plant J 32: 775–787. 12472692 10.1046/j.1365-313x.2002.01468.x

[36] Nevoigt E, Fassbender A, Stahl U (2000) Cells of the yeast Saccharomyces cerevisiae are transformable by DNA under non-artificial conditions. Yeast 16: 1107–1110. 10953082 10.1002/1097-0061(20000915)16:12<1107::AID-YEA608>3.0.CO;2-3

[37] Moriguchi K, Edahiro N, Yamamoto S, Tanaka K, Kurata N, et al (2013) Trans-kingdom genetic transfer from Escherichia coli to Saccharomyces cerevisiae as a simple gene introduction tool. Appl Environ Microbiol 79: 4393–4400. 23666333 10.1128/AEM.00770-13 PMC3697487

[38] Yamamoto S, Uraji M, Tanaka K, Moriguchi K, Suziki K (2007) Identification of pTi-SAKURA DNA region conferring enhancement of plasmid incompatibility and stability. Genes Genet Syst 82: 197–206. 17660690 10.1266/ggs.82.197

[39] Nishikawa M, Suzuki K, Yoshida K (1992) DNA integration into recipient yeast chromosomes by trans-kingdom conjugation between Escherichia coli and Saccharomyces cerevisiae. Curr Genet 21: 101–108. 1568253 10.1007/BF00318467

[40] Pansegrau W, Lanka E, Barth PT, Figurski DH, Guiney DG, et al (1994) Complete nucleotide sequence of Birmingham IncP alpha plasmids, Compliation and comparative analysis. J Mol Biol 239: 623–663. 8014987 10.1006/jmbi.1994.1404

[41] Nishikawa M, Suzuki K, Yoshida K (1990) Structural and functional stability of IncP plasmids during stepwise transmission by trans-kingdom mating: promiscuous conjugation of Escherichia coli and Saccharomyces cerevisiae. Jpn J Genet 65: 323–334. 2248784 10.1266/jjg.65.323

[42] Nishikawa M, Yoshida K (1998) Trans-kingdom conjugation offers a powerful gene targeting: tool in yeast. Genet Anal 14: 65–73. 9526697 10.1016/s1050-3862(97)10003-1

