# Correction: Trans-Kingdom Horizontal DNA Transfer from Bacteria to Yeast Is Highly Plastic Due to Natural Polymorphisms in Auxiliary Nonessential Recipient Genes

**DOI:** 10.1371/journal.pone.0126431

**Published:** 2015-04-13

**Authors:** The PLOS ONE Staff

The fourth and fifth sentences of the Materials and Methods section are incorrect. The correct sentences are: TNB (80 mM Tris-HCl (pH 7.5) and 0.05% NaCl) was used for the standard TKC reaction. Alternatively, TNB at pH 6.5, MNB (80 mM MES (pH 5.5) and 0.05% NaCl), and PBS (purchased from Becton, Dickinson and Company; adjusted to pH 7.5) were used for analyzing the adaptability of TKC under various buffers.
