# Supplementary figures and images for: Trans-Kingdom Horizontal DNA Transfer from Bacteria to Yeast Is Highly Plastic Due to Natural Polymorphisms in Auxiliary Nonessential Recipient Genes
Source: PLoS One. 2013 Sep 13;8(9):e74590. doi: 10.1371/journal.pone.0074590 (PMC3772842; doi:10.1371/journal.pone.0074590)

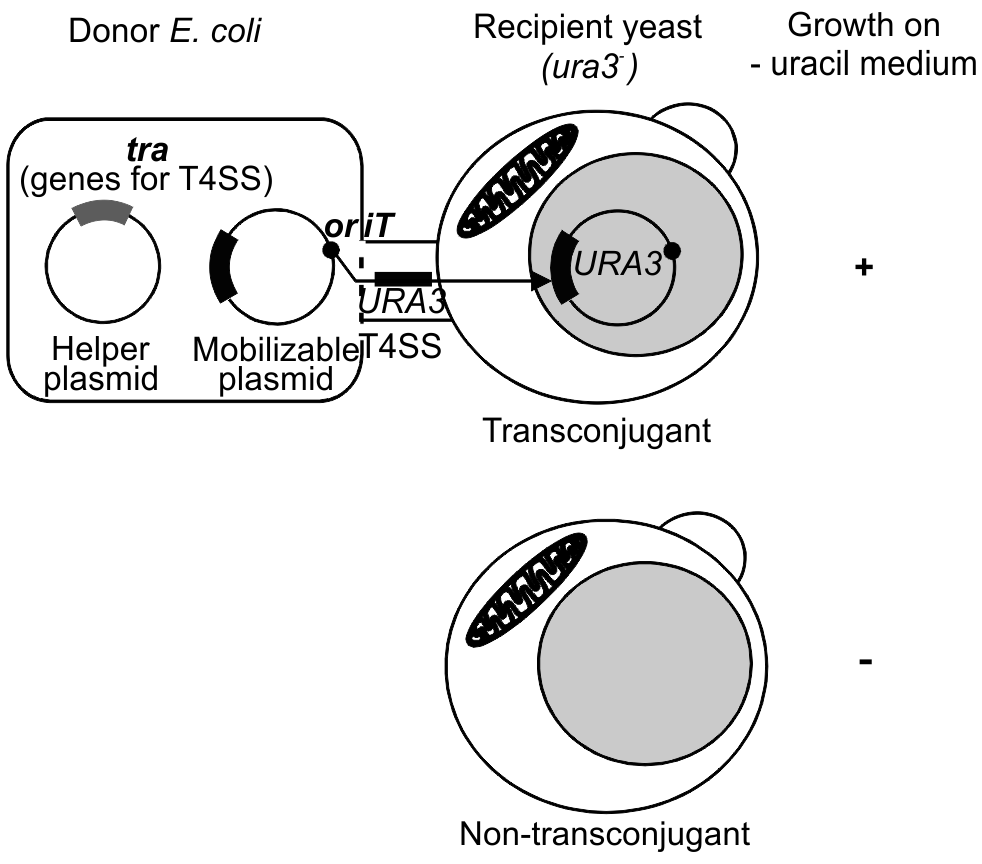

Supplement: Figure S1 — Schematic representation of TKC detection. The donor E. coli has a helper plasmid and a mobilizable plasmid. The helper plasmid contains T4SS genes derived from an IncP1α plasmid, called tra genes (gray box). The mobilizable plasmid contains the origin of transfer (oriT; black dot) of an IncQ plasmid and URA3 gene (black box). When the mobilizable plasmid transfers into the recipient yeast cell lacking the URA3 gene, the transconjugant survives on selection medium with no added uracil. (TIF) [file pone.0074590.s001.tif]

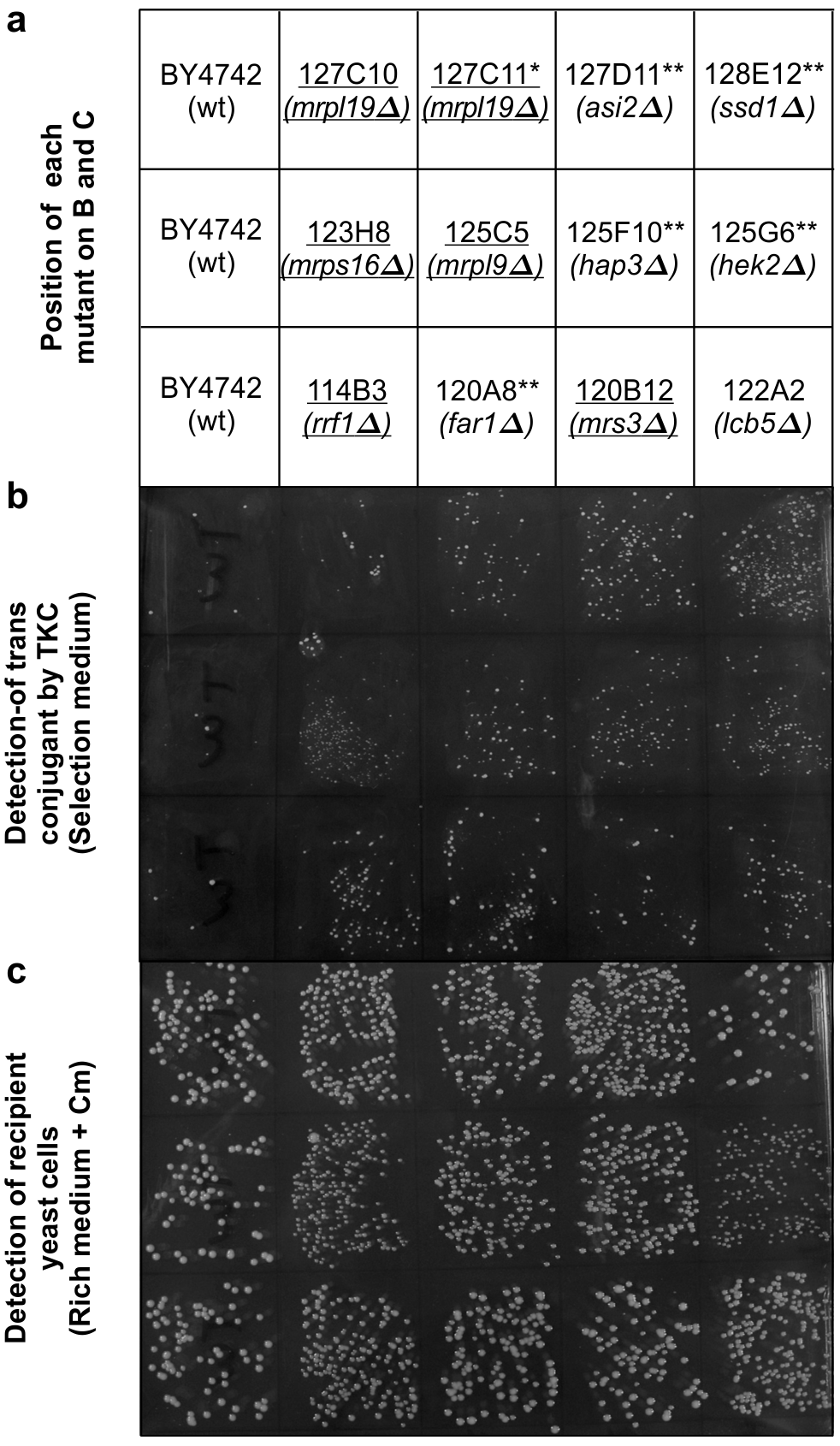

Supplement: Figure S2 — An example of the TKC results at the third screening. (A) Template showing format of plating of the various mutants. Mutants for nuclear-encoded mitochondrial genes are underlined. * KO mutant of dubious ORF unlikely to encode a protein, and its overlapping gene is shown in parentheses. ** High-receptivity mutants included in Table S1. (B) Transconjugants grown on a selection plate; volume equivalent to 50% of each TKC reaction mix was plated. (C) Recipient yeast cells grown on a YPD+chloramphenicol plate; a volume equivalent to 1/5000 of each TKC reaction mix was plated. HB101-containing plasmids pRH210 and pAY205 were used as the donors. (TIF) [file pone.0074590.s002.tif]

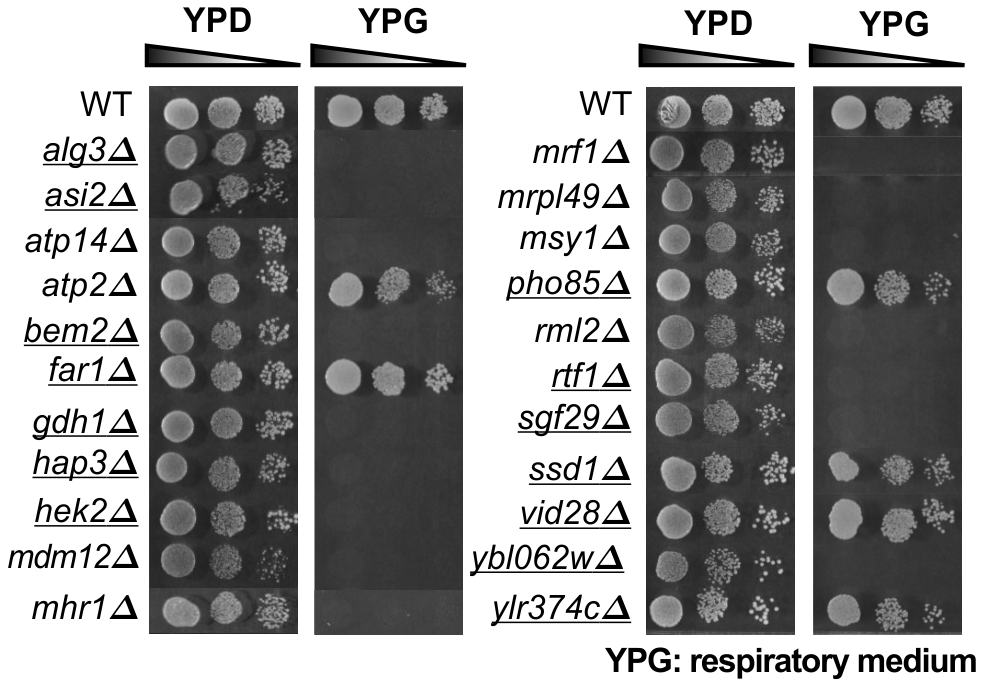

Supplement: Figure S3 — Confirmation of the mitochondrial integrity among the identified high-receptivity mutants. The high-receptivity mutants were mated with a MATa strain, carrying wild-type nuclear genome but a rho 0 mitochondrial genome derived from the BY4741 strain. The resultant heterogeneous diploid strains were serially diluted and spotted on both rich glucose (YPD) and rich respiratory glycerol (YPG) media, and were incubated at 28°C for 48 h and 72 h, respectively. The 14 KO mutants for non-mitochondrial genes are underlined. (TIF) [file pone.0074590.s003.tif]

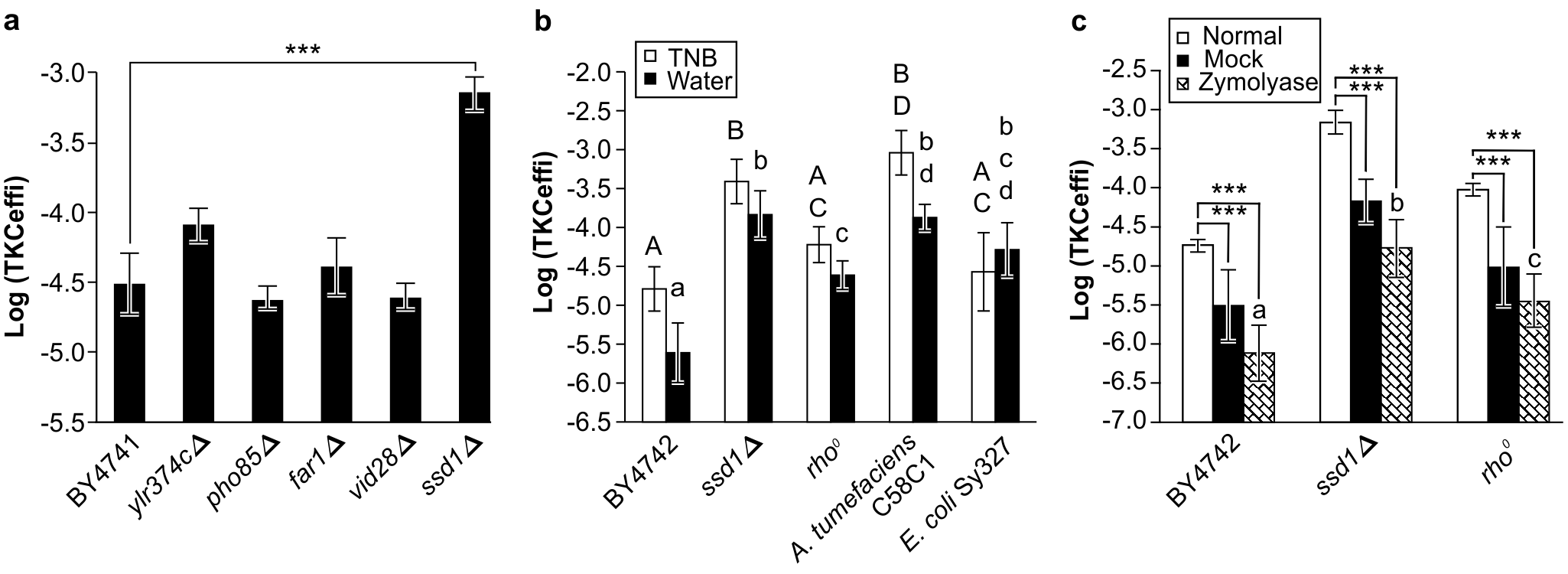

Supplement: Figure S4 — Confirmation and characterization of the high-receptivity mutants. (A) Effect of knock-out mutation in the 5 candidate genes in the yeast strain BY4741 on TKC. Data are represented as mean ± SD (n = 3). Asterisks indicate a statistically significant difference: ***p<0.001 (two-tailed t-test). (B) Comparison between TKC and bacterial conjugation efficiency under TNB and filter-sterilized freshwater environments. Data are represented as mean ± SD (n = 5). Upper- and lowercase letters above the bars indicate significant differences at p<0.05 (Holm’s test) among yeast and bacterial strains under each condition. (C) Effect of cell wall digestion on TKC efficiency. Normal: the recipient cells were resuspended in TNB before testing for TKC. Mock: cells pretreated with TNB +1 M sorbitol, and TKC reaction performed in the same solution. Zymolyase: pretreatment with TNB +1 M sorbitol +0.5 mg/mL Zymolyase-100 T, and TKC reaction performed in TNB +1 M sorbitol. Each pretreatment was performed for 1 h at 28°C. Data are represented as mean ± SD (n = 7 in BY4742 with sorbitol, n = 4 in others). Lowercase letters above the bars indicate significant differences at p<0.05 (Holm’s test). HB101 (pRH210, pAY205) was used as the donor in all experiments. (TIF) [file pone.0074590.s004.tif]

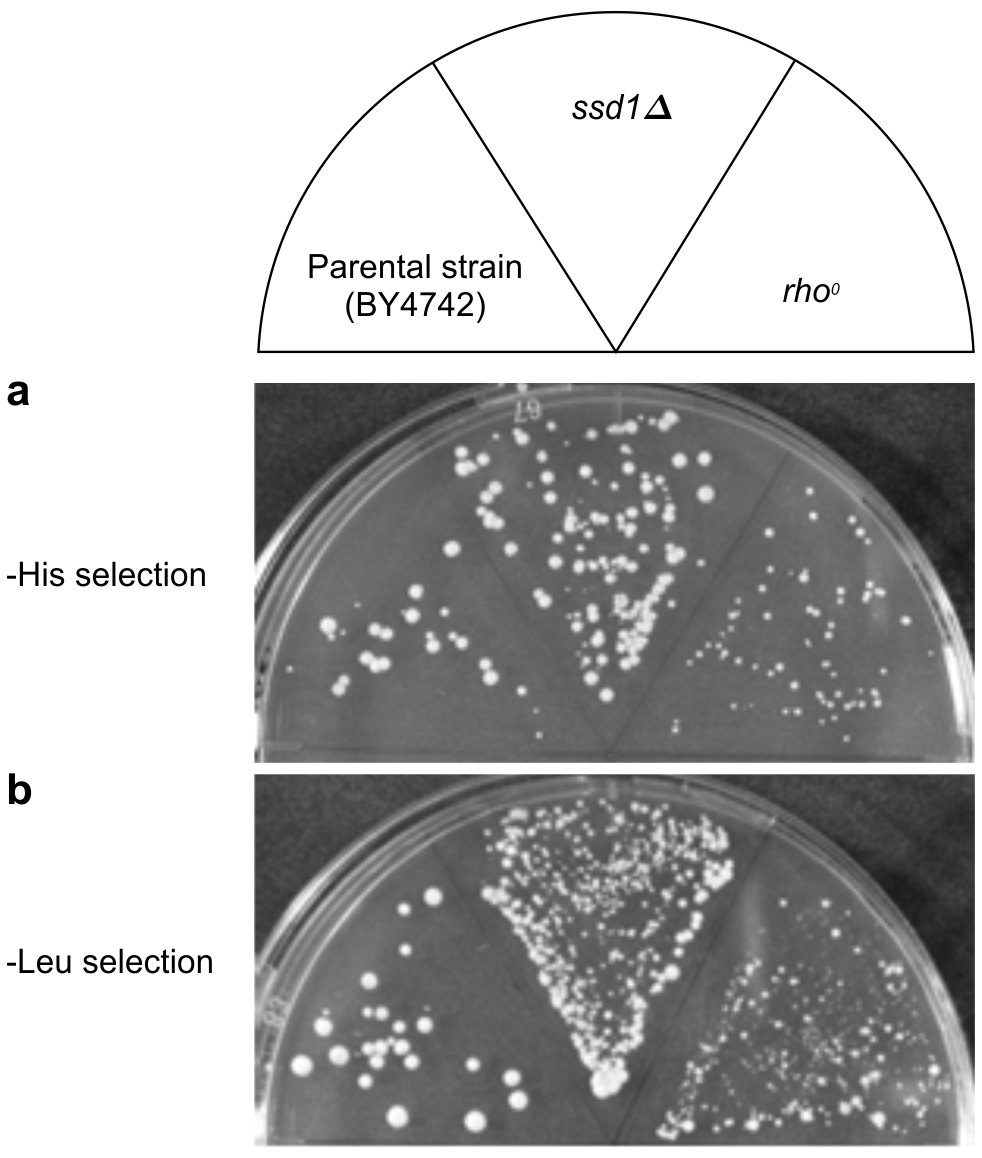

Supplement: Figure S5 — Confirmation of high TKC receptivity in ssd1Δ and rho 0 mutants using other selection markers. (A) A TKC vector pRS313::oriT P, carrying HIS3 gene as a selection marker, was used and the transconjugants in parental and mutant strains were selected on a selection medium plate lacking leucine. (B) A TKC vector pRS315::oriT P, carrying HIS3 gene as a selection marker, was used. (TIF) [file pone.0074590.s005.tif]
