# Supplementary material for: Trans-Kingdom Horizontal DNA Transfer from Bacteria to Yeast Is Highly Plastic Due to Natural Polymorphisms in Auxiliary Nonessential Recipient Genes
Source: PLoS One. 2013 Sep 13;8(9):e74590. doi: 10.1371/journal.pone.0074590 (PMC3772842; doi:10.1371/journal.pone.0074590)
Supplement: Table S1 — List of high-receptivity mutants screened from the complete set of Yeast Deletion Clones ( MATα haploids complete set). Efficiency of transfer of the URA3 marker gene from Escherichia coli to various yeast deletion strains was measured relative to transfer to the parental strain (fold increase vs. wt). (DOC) [file pone.0074590.s006.doc]

**Table S1.** List of high-receptivity mutants screened from the complete set of Yeast Deletion Clones (*MATα* haploids complete set). Efficiency of transfer of the *URA3* marker gene from *Escherichia coli* to various yeast deletion strains was measured relative to transfer to the parental strain (fold increase vs. wt).

| Strains | Knock-out genes | | TKC efficiency | | Chemical transformation efficiency |
| --- | --- | --- | --- | --- | --- |
|  | Systematic name | Standard name | 2^nd^ screening | 3^rd^ screening |  |
| 109E4^a^ | *YPL097W* | *MSY1*^c^ | 12.3 | 28.6 | 0.5 |
| 112B9^a^ | *YEL050C* | *RML2*^c^ | 18.2 | 31.1 | 0.7 |
| 120A8 | *YJL157C* | *FAR1* | 19.4 | 44.2 | 0.8 |
| 122C7^a^ | *YLR295C* | *ATP14*^c^ | 17.3 | 25.8 | 0.4 |
| 122H2^a^ | *YGL244W* | *RTF1* | 10.6 | 28.9 | 0.2 |
| 123G5 | *YPL031C* | *PHO85* | 33.9 | 37.8 | 0.6 |
| 125F10^a^ | *YBL021C* | *HAP3* | 16.6 | 17.3 | 0.4 |
| 125G6^a^ | *YBL032W* | *HEK2* | 8.8 | 19.2 | 0.3 |
| 126A7^a^ | *YBL062W* ^b^ | *(SKT5)* | 19.6 | 12.8 | 1.7 |
| 126B11^a^ | *YBL082C* | *ALG3* | 18.3 | 17.5 | 0.7 |
| 127D11^a^ | *YNL159C* | *ASI2* | 22.0 | 13.1 | 1.0 |
| 128E12 | *YDR293C* | *SSD1* | 18.6 | 74.9 | 1.6 |
| 128F3^a^ | *YDR296W* | *MHR1*^c^ | 17.5 | 6.7 | 0.8 |
| 129A7 | *YIL017C* | *VID28* | 17.4 | 11.4 | 0.6 |
| 136C5^a^ | *YJR121W* | *ATP2*^c^ | 8.0 | 20.8 | 0.7 |
| 148B1^a^ | *YGL143C* | *MRF1*^c^ | 11.3 | 16.6 | 1.1 |
| 148H2^a^ | *YER155C* | *BEM2* | 9.0 | 30.2 | 0.5 |
| 149E6 | *YLR374C* ^b^ | *(STP3)* | 8.6 | 24.6 | 0.9 |
| 170C9^a^ | *YOR375C* | *GDH1* | 21.4 | 13.8 | 0.7 |
| 170C10^a^ | *YOL009C* | *MDM12*^c^ | 19.0 | 18.7 | 0.2 |
| 170E9^a^ | *YCL010C* | *SGF29* | 33.8 | 43.4 | 0.8 |
| 171B9^a^ | *YJL096W* | *MRPL49*^c^ | 16.3 | 9.2 | 0.5 |

^a^Petite mutants. ^b^Dubious ORFs unlikely to encode proteins, and their overlapping genes are shown in parentheses. ^c^Nuclear encoded mitochondrial genes.

Average values are shown (n = 3).
