# Supplementary material for: Trans-Kingdom Horizontal DNA Transfer from Bacteria to Yeast Is Highly Plastic Due to Natural Polymorphisms in Auxiliary Nonessential Recipient Genes
Source: PLoS One. 2013 Sep 13;8(9):e74590. doi: 10.1371/journal.pone.0074590 (PMC3772842; doi:10.1371/journal.pone.0074590)
Supplement: Table S2 — Results of the TKC experiment without the helper plasmid. (DOC) [file pone.0074590.s007.doc]

|  | Recipient | Exp. 1 | Exp. 2 | Exp. 3 | Exp. 4 | Average |
| --- | --- | --- | --- | --- | --- | --- |
| TKC efficiency  (× 10^-7^) | Wild-type | < 5.8 | < 7.0 | < 7.1 | < 6.4 | < 6.6 |
|  | *ssd1Δ* | < 5.7 | < 6.5 | < 5.5 | < 5.7 | < 5.9 |
|  | *rho*^0^ | < 4.0 | < 4.0 | < 3.7 | < 4.6 | < 4.1 |
| Log_10_(TKCeffi) | Wild-type | < -6.2 | < -6.2 | < -6.2 | < -6.2 | < -6.2 |
|  | *ssd1Δ* | < -6.2 | < -6.2 | < -6.3 | < -6.2 | < -6.2 |
|  | *rho*^0^ | < -6.4 | < -6.4 | < -6.4 | < -6.3 | < -6.4 |

**Table S2.** Results of the TKC experiment without the helper plasmid.

Cells of *E. coli* HB101 carrying a mobilizable plasmid, pAY205 (1.1 × 10^7^), were incubated with recipient yeast strains (3.0 × 10^6^ cells) in a trans-kingdom conjugation reaction. The donor strain lacked the helper plasmid carrying a T4SS gene. As no transconjugant was obtained, TKC efficiency (transconjugants/recipient cell) and Log_10_-converted TKC efficiency were estimated to the maximum value, supposing that 1 transconjugant was isolated in each experiment.
