# Supplementary material for: Trans-Kingdom Horizontal DNA Transfer from Bacteria to Yeast Is Highly Plastic Due to Natural Polymorphisms in Auxiliary Nonessential Recipient Genes
Source: PLoS One. 2013 Sep 13;8(9):e74590. doi: 10.1371/journal.pone.0074590 (PMC3772842; doi:10.1371/journal.pone.0074590)
Supplement: Table S3 — PCR primers used in this study. (DOC) [file pone.0074590.s008.doc]

**Table S3.** PCR primers used in this study.

| Target gene | Primer name | Sequence |
| --- | --- | --- |
| *YLR374C* | YLR374C (STP3) FW | 5′-TGCGTCGACCGTGGTCAGATTGGCATA-3′ |
|  | YLR374C (STP3) RV | 5′-TTAGTCGACTCGGTTGGGTCCATTAG-3′ |
|  | YLR374C outer FW | 5′-GGTGTCGCAACGTGAG-3′ |
|  | YLR374C inner FW | 5′-ATGTTCATATTAGGCAGTGTCG-3′ |
|  | YLR374C inner RV | 5′-TTATTGTTTGCTGATCCCA-3′ |
| *PHO85* | PHO85 FW | 5′-ACAGTCGACTCACCGCTTTCATTACCA-3′ |
|  | PHO85 RV | 5′-AGGGTCGACAACCAGGGAACGAACCGATA-3′ |
|  | PHO85 outer FW | 5′-ATTCATGAACGGGTGA-3′ |
|  | PHO85 inner FW | 5′-CTGCAAGGGCTGGC-3′ |
|  | PHO85 inner RV | 5′-GCGTTATGAAGCGTGGTG-3′ |
| *FAR1* | FAR1 FW | 5′-GGGGTCGACGGCGCATGGATCTTGACC-3′ |
|  | FAR1 RV | 5′-GCGGTCGACGGCCCGTGGAAATCGTAT-3′ |
|  | FAR1 outer FW | 5′-GGGCCCGACGGAAAGTTA-3′ |
|  | FAR1 inner FW | 5′-GAGCGCCGCCTGATGTTA-3′ |
|  | FAR1 inner RV | 5′-TCACCCGCAGCCATATCC-3′ |
| *VID28* | VID28 FW | 5′-CAAGGATCCATTGCGTTGTGCGACATTA-3′ |
|  | VID28 RV | 5′-GTGGGATCCCCGGTGGTGGTAGAGTTGA-3′ |
|  | VID28 outer FW | 5′-CCTTTATGACGCCTTTCCT-3′ |
|  | VID28 inner FW | 5′-CAGAACGGTTCATCCAG-3′ |
|  | VID28 inner RV | 5′-CGCGTACTTCCCATTTC-3′ |
| *SSD1* | SSD1 FW | 5′-CATGTCGACATTAAAGGCCACGAACAGG-3′ |
|  | SSD1 RV | 5′-AAAGTCGACCCGTCTGTATTTGCGTTTGC-3′ |
|  | SSD1 outer FW | 5′-CCTATGTCACGGACGA-3′ |
|  | SSD1 inner FW | 5′-ACGTTGGCCATCACAT-3′ |
|  | SSD1 inner RV | 5′-TCGCCTACCCAAGTAGAT-3′ |
